# Supplementary material for: Psychological Distress Associated with Enforced Hospital Isolation Due to COVID-19 during the “Flatten the Curve” Phase in Morocco: A Single-Center Cross-Sectional Study
Source: Healthcare (Basel). 2024 Feb 26;12(5):548. doi: 10.3390/healthcare12050548 (PMC10930375; doi:10.3390/healthcare12050548)
Supplement: Supplementary file 1 [file healthcare-12-00548-s001.zip › healthcare-2767455-supplementary.pdf]

Supplementary File

Figure S1: Hospital Anxiety and Depression Scale (HADS)

Hospital Anxiety and Depression Scale (HADS)

Tick the box beside the reply that is closest to how you have been feeling in the past week.
Don't take too long over you replies: your immediate is best.

D	A		D	A	
		**I feel tense or 'wound up':**			**I feel as if I am slowed down:**
	3	Most of the time	3		Nearly all the time
	2	A lot of the time	2		Very often
	1	From time to time, occasionally	1		Sometimes
	0	Not at all	0		Not at all
		**I still enjoy the things I used to enjoy:**			**I get a sort of frightened feeling like 'butterflies' in the stomach:**
0		Definitely as much		0	Not at all
1		Not quite so much		1	Occasionally
2		Only a little		2	Quite Often
3		Hardly at all		3	Very Often
		**I get a sort of frightened feeling as if something awful is about to happen:**			**I have lost interest in my appearance:**
	3	Very definitely and quite badly	3		Definitely
	2	Yes, but not too badly	2		I don't take as much care as I should
	1	A little, but it doesn't worry me	1		I may not take quite as much care
	0	Not at all	0		I take just as much care as ever
		**I can laugh and see the funny side of things:**			**I feel restless as I have to be on the move:**
0		As much as I always could		3	Very much indeed
1		Not quite so much now		2	Quite a lot
2		Definitely not so much now		1	Not very much
3		Not at all		0	Not at all
		**Worrying thoughts go through my mind:**			**I look forward with enjoyment to things:**
	3	A great deal of the time	0		As much as I ever did
	2	A lot of the time	1		Rather less than I used to
	1	From time to time, but not too often	2		Definitely less than I used to
	0	Only occasionally	3		Hardly at all
		**I feel cheerful:**			**I get sudden feelings of panic:**
3		Not at all		3	Very often indeed
2		Not often		2	Quite often
1		Sometimes		1	Not very often
0		Most of the time		0	Not at all
		**I can sit at ease and feel relaxed:**			**I can enjoy a good book or radio or TV program:**
	0	Definitely	0		Often
	1	Usually	1		Sometimes
	2	Not Often	2		Not often
	3	Not at all	3		Very seldom

Please check you have answered all the questions

Scoring:

Total score: Depression (D) ___________ Anxiety (A) _____________

0-7 = Normal

8-10 = Borderline abnormal (borderline case)

11-21 = Abnormal (case)
